# Root symbionts alter herbivore-induced indirect defenses of tomato plants by enhancing predator attraction

**DOI:** 10.3389/fphys.2022.1003746

**Published:** 2022-10-21

**Authors:** Dimitra Papantoniou, Dongik Chang, Ainhoa Martínez-Medina, Nicole M. van Dam, Alexander Weinhold

**Affiliations:** ^1^ German Centre for Integrative Biodiversity Research (iDiv) Halle-Jena-Leipzig, Leipzig, Germany; ^2^ Institute of Biodiversity, Friedrich-Schiller Universität Jena, Jena, Germany; ^3^ Plant-Microorganism Interaction, Institute of Natural Resources and Agrobiology of Salamanca, Salamanca, Spain

**Keywords:** arbuscular mycorrhizal fungi, *Trichoderma*, *Spodoptera exigua*, *Macrolophus pygmaeus*, GC-MS, herbivore-induced plant volatiles, multi-trophic interactions

## Abstract

Beneficial root microbes are among the most frequently used biocontrol agents in cropping systems, since they have been shown to promote plant growth and crop yield. Moreover, they are able to enhance protection against pathogens and insect herbivores by activating plant resistance mechanisms. Plant defense responses against herbivorous insects include the induction of metabolic pathways involved in the synthesis of defense-related metabolites. These metabolites include volatile organic compounds (VOCs), which attract natural enemies of the herbivores as a form of indirect resistance. Considering that beneficial root microbes may affect direct herbivore resistance, we hypothesized that also indirect resistance may be affected. We tested this hypothesis in a study system composed of tomato, the arbuscular mycorrhizal fungus *Rhizophagus irregularis*, the growth-promoting fungus *Trichoderma harzianum*, the generalist chewing herbivore *Spodoptera exigua* and the omnivorous predator *Macrolophus pygmaeus*. Using a Y-tube olfactometer we found that *M. pygmaeus* preferred plants with *S. exigua* herbivory, but microbe-inoculated plants more than non-inoculated ones. We used a targeted GC-MS approach to assess the impact of beneficial microbes on the emission of volatiles 24 h after herbivory to explain the choice of *M. pygmaeus*. We observed that the volatile composition of the herbivore-infested plants differed from that of the non-infested plants, which was driven by the higher emission of green leaf volatile compounds, methyl salicylate, and several monoterpenes and sesquiterpenes. Inoculation with microbes had only a marginal effect on the emission of some terpenoids in our experiment. Gene expression analysis showed that the marker genes involved in the jasmonic and salicylic acid pathways were differentially expressed in the microbe-inoculated plants after herbivory. Our results pinpoint the role of root symbionts in determining plant-microbe-insect interactions up to the third trophic level, and elucidates their potential to be used in plant protection.

## Introduction

Plants encounter multiple biotic and abiotic challenges simultaneously in their habitats (Walling, 2000). Among the biotic challenges they face, insect pests represent one of the most important factors, leading to extensive agricultural crop losses. According to recent studies, on average, pests can destroy up to 40% of global crop production (Savary et al., 2019; IPPC Secretariat, 2021). To counter herbivore attack, plants mount a wide array of defense mechanisms, which include specialized morphological structures or secondary metabolites and proteins that have toxic, repellent and/or anti-nutritional effects on herbivores (Usha Rani and Jyothsna, 2010; War et al., 2012). Defense mechanisms that affect host plant preference, survival or reproductive success are characterized as direct defenses, whereas mechanisms that involve natural enemies of the attacking pests are characterized as indirect defenses (Turlings and Erb, 2018). One form of indirect plant defenses in response to insect herbivory is the release of a bouquet of Herbivore-Induced Plant Volatiles (HIPVs) that specifically attract natural enemies of the attacking herbivores (Arimura et al., 2009; McCormick et al., 2012). The blends of HIPVs emitted in response to herbivore attack predominantly include terpenes, green leaf volatiles (GLVs) and methyl salicylate (MeSA) (Dudareva et al., 2006; Maffei, 2010). HIPV emission results from the activation of specific genes, which are involved mainly into the terpenoid, lipoxygenase, and shikimic acid pathways (Dudareva et al., 2006; Pichersky et al., 2006; Maffei, 2010). These so-called info-chemicals serve several roles, such as the interaction of plants with arthropods, microorganisms, undamaged neighboring plants, or intra-plant signaling that warns undamaged parts within the plant under attack (Karban, 2011; Turlings and Erb, 2018). Therefore, HIPVs can be effectively used in crop pest management.

In the last decades, management of crop pests has heavily relied on conventional insecticides resulting in reduced crop losses by pests. However, the intensive and large-scale application of insecticides has caused several adverse effects on the environment. Persistent pesticides are found in soils and water, which affects not only target but also of non-target species (Bragança et al., 2018). Moreover, the adaptation and increased resistance to pesticides developed by the target pests has led to the need of using higher amounts and new chemical compounds to protect crops every year, leading to undesired side effects and increasing the costs of food production (Carvalho, 2006). Integrated Pest Management (IPM) is an effective, environmentally sound approach to pest management (Kabir and Rainis, 2015). IPM strategies aim at protecting air, water and soil resources and employ a variety of pest-control methods in a way that facilitates biological control of insect pests in order to improve economic, public health and environmental outcomes (Alam et al., 2016).

In their habitats, plants not only have detrimental but also symbiotic interactions with several growth promoting microbes. Such microbes provide plants with an improved nutritional status (Hodge and Fitter, 2010; Samolski et al., 2012); tolerance to biotic and abiotic stresses (Fontenelle et al., 2011; Degola et al., 2015) and improved fruit yield and quality (Hermosa et al., 2012; Bona et al., 2018). In the last decades, root beneficial microbes have also been included in IPM programs in an effort to enhance the efficacy of pest control, reduce the use of synthetic chemicals, increase crop yield and improve the quality of products (Woo et al., 2014).

A well-documented example of plant-associated beneficial microbes are the arbuscular mycorrhizal fungi (AMF). AMF are symbiotic inhabitants of plant roots and form associations with over 80% of the terrestrial plant species (Parniske, 2008). They are able to enhance plant growth and development by improving the acquisition of water and mineral nutrients, such as inorganic phosphate and various micronutrients (Smith and Smith, 2011; Hodge and Storer, 2015). Moreover, mycorrhizal plants have shown higher tolerance to abiotic stress agents (Miransari, 2010; Smith et al., 2010; Orine et al., 2022) and increased resistance to plant pests and diseases through the induction of systemic resistance (Pozo and Azcón-Aguilar, 2007; Pineda et al., 2010; Jung et al., 2012). In exchange, AMF obtain plant-derived photoassimilates (Bago et al., 2000; Smith and Read, 2010) produced during photosynthesis. Although the fungal structures formed during mycorrhizal symbiosis are restricted in the plant roots, AMF trigger systemic biochemical changes in the composition and concentration of primary and secondary metabolites in the aboveground plant tissues (Schweiger et al., 2014; Schweiger and Müller, 2015). Such metabolic changes influence not only the performance of the insect herbivores feeding on the host plants (Papantoniou et al., 2021), but also their interactions with their natural enemies (Minton et al., 2016). Studies have shown that arbuscular mycorrhizal fungi can modulate the indirect plant defenses and the recruitment of natural enemies of herbivorous pests as well (Fontana et al., 2009; Schausberger et al., 2012; Babikova et al., 2014).

In the broad spectrum of plant-associated microbes, various strains of the soil-borne fungus *Trichoderma* have direct beneficial effects on plants including the promotion of growth and nutrient uptake, better assimilation of nitrogen and enhancement of plant defenses against abiotic and biotic stress factors (Shoresh et al., 2010; Studholme et al., 2013; Lorito and Woo, 2015). *Trichoderma* strains have been reported to alter herbivore-induced plant volatiles and the expression levels of genes involved in the induction of indirect defense responses resulting in a stronger attraction of natural enemies towards the herbivorous pests (Battaglia et al., 2013; Coppola et al., 2017; Coppola et al., 2019).

Tomato (*Solanum lycopersicum* L.) is the second most cultivated vegetable crop throughout the world and susceptible to numerous pests that can severely affect its nutritional value and taste (Zhang et al., 2021). Among them are the larval stages of *Spodoptera exigua* (Hübner), the beet armyworm, which is a polyphagous insect with a wide global distribution (Moulton et al., 2000; Wang et al., 2006; Zheng et al., 2011). Therefore, IPM strategies combining the use of root symbionts and natural enemies to prey on *S. exigua* larvae could be a promising approach to control tomato pests in greenhouse and field crops.

Hemipteran predators of the Miridae family are commonly used as natural enemies to control pests. Recent studies suggest that these mirid predators use HIPVs as cues to locate their prey (Moayeri et al., 2007; Lins et al., 2014; Silva et al., 2018). Use of such info-chemicals has been observed among many predaceous arthropods independently of their diet specialization (Dicke and Sabelis, 1988); however, learning to respond to info-chemicals occurs more commonly in generalists than in specialists (Steidle and Van Loon, 2003). The zoophytophagous mirid *Macrolophus pygmaeus* Rambur (Hemiptera: Miridae) is broadly used as a biocontrol agent to control greenhouse and field tomato pests in Europe (Castañé et al., 2004; Perdikis et al., 2008; Arnó et al., 2010). The predator is mass-reared and released to control thrips, whiteflies, mites and lepidopterans, but can also prey on many other pest species (Van Lenteren et al., 2020). *M. pygmaeus* reacts to volatiles emitted by prey-infested plants, but not to volatiles emitted directly by the prey (Moayeri H. et al., 2006; Moayeri H. R. et al., 2006; Ingegno et al., 2011; Mollá et al., 2014).

So far, few studies focusing on the effect of root symbionts on the induction of indirect defenses of tomato plants against herbivores and the attraction of their natural enemies have been conducted. The majority has cast light on the effect of tomato root-associated microbes on the emission of volatiles after aphid infestation and their contribution to attracting aphid predators or parasitoids (Guerrieri et al., 2004; Battaglia et al., 2013; Coppola et al., 2017). Only the study of (Coppola et al., 2019) investigated how the biocontrol agent *Trichoderma atroviride* strain P1 induces tomato plant responses against two insects with different feeding habits, the noctuid caterpillar *Spodoptera littoralis* and the aphid *Macrosiphum euphorbiae*. They found that *T. atroviride* P1 altered the plant metabolic pathways leading to the production and emission of VOCs, which were linked to the higher attraction of the parasitoid *Aphidius ervi*. This indicated that root symbionts might affect the response of natural enemies to the plant. However, they tested the attraction of the parasitoid, toward *T. atroviride* P1-inoculated and untreated plants without prior exposure to the herbivores and then collected the volatiles from the plants used in the wind-tunnel bioassays.

To widen our knowledge of the effect of root symbionts on indirect defenses, we set up a series of experiments with tomato plants root-inoculated with two different beneficial microbes, the arbuscular mycorrhizal fungus *Rhizophagus irregularis* or the plant growth-promoting fungus *Trichoderma harzianum.* We exposed the plants to herbivory by the generalist lepidopteran herbivore *S. exigua* and assessed the choice behavior of the zoophytophagous mirid predator *M. pygmaeus*. We hypothesized that inoculation of plants with root symbionts would enhance the indirect defense responses of tomato in response to herbivory, resulting in higher expression of the genes involved in the biosynthesis of herbivore-induced plant volatiles and therefore, a stronger attraction of *M. pygmaeus*. To test our hypotheses, we used a Y-tube olfactometer to test the preference of *M. pygmaeus* towards microbe-inoculated and non-inoculated plants with and without the herbivore. To identify potential mechanisms driving *M. pygmaeus* preferences, we collected volatiles emitted by non-inoculated and microbe-inoculated plants in the presence and absence of aboveground herbivory. We complemented our study with gene expression analysis of specific genes, which are involved in the biosynthetic pathways that regulate the synthesis of herbivore-induced plant volatiles. These complementary analyses allowed us to generate some more insights in the interactions between root symbiotic fungi and natural control agents commonly used in tomato culture.

## Materials and methods

### Plant and fungal material

Tomato (*Solanum lycopersicum*) cultivar Moneymaker was used in all the bioassays as well as in the predator rearing. Tomato seeds were purchased from Intratuin B.V (Woerden, Netherlands). *Rhizophagus irregularis* was used as the AM fungus. The *R. irregularis* solid inoculum (DAOM197198 research grade, 1 million spores in 100 g attapulgite powder, batch S.380—02.2021) and the heat-sterilized carrier material were purchased from SYMPLANTA GmbH & Co. KG (Darmstadt, Germany, https://www.symplanta.com) and stored at 8°C until used. *Trichoderma harzianum* isolate T-78 (CECT 20714, Spanish Type Culture Collection) was routinely cultured on potato dextrose agar (PDA, Sifin Diagnostics, Berlin, Germany) plates and regularly sub-cultured. The *T. harzianum* inoculum was prepared on a solid medium containing commercial oat and vermiculite according to Martínez-Medina et al. (2009).

### Insect rearing

Eggs of *S. exigua* (Lepidoptera, Noctuidae) were obtained from Entocare Biologische Gewasbescherming (Wageningen, Netherlands, www.entocare.nl). The colony of *S. exigua* was routinely maintained in a growth chamber (E-36L, Percival Scientific, Perry, United States) at 25°C, 12 h L, and 12 h D, 45% RH conditions and fed upon artificial diet (Hoffman et al., 1966). Three hundred adults of *M. pygmaeus* were purchased from Katz Biotech AG (Baruth, Germany) and reared in the laboratory reaching adult developmental stage in the next generation. The insects were reared in net cages containing young tomato plants that were grown exclusively for this purpose. The omnivorous insects were provided *ad libitum* with supplementary food including *Sitotroga* eggs (Katz Biotech AG, Baruth, Germany), 1:4 diluted honey water (v/v) soaked on a piece of cotton pad, and organic bee pollen (Biojoy GmbH, Nürnberg, Germany) as described by Vandekerkhove and De Clercq (2010). The insect cages were placed in a plant growth chamber (E-36L, Percival Scientific, Perry, United States) at 24°C with 14 h L and at 21°C with 10 h D and 60% RH conditions. The plants used for *M. pygmaeus* rearing in the net cage were provided with tap water every 2 days and fertilized with Hoagland nutrient solution (Hoagland and Arnon, 1950) once per week.

### Plant growth conditions and fungal inoculation

Tomato seeds were surface-sterilized with 40 ml of 10% (v/v) sodium hypochlorite (NaOCL, 12% ChemSolute, Th. Geyer, Berlin, Germany) for 3 min, then thoroughly rinsed in 40 ml of warm tap water repeatedly for four times. After sterilization, the seeds were placed on fine-grained, moist vermiculite and germinated in the dark for 3 days at 28°C. Subsequently, the germinated seedlings were placed in a plant growth chamber (E-36L, Percival Scientific, Perry, United States) at 24 ± 3°C, 14 h/10 h L/D and 60–65% RH conditions. Ten days after germination, the seedlings were transplanted into 1 L pots containing a sterile soil/sand mixture (1:1, v/v). Inoculation with *R. irregularis* was achieved by applying 30 AMF spores per 1 ml of sterile potting substrate mixture directly to the root while potting in order to achieve higher initial concentrations than homogenizing the soil with the inoculum. Inoculation with *T. harzianum* was achieved by mixing the *T. harzianum* inoculum with the sterile potting substrate to achieve a final density of 1 × 10^6^ conidia g^−1^ before transplanting (Martínez-Medina et al., 2017). The plants were then placed in a greenhouse (3.8 m × 6 m) in a completely randomized design with supplemental LED lighting of 3500 k and 80 CRI (RUBOL JOSEPHINE 135W V2 LUMINUS CXM-32 DIY KIT, Rubol, Dronten, Netherlands), 16 h/8 h L/D and ventilation provided within 10 min intervals. The air temperature and relative humidity in the greenhouse were recorded while the experiment took place. The recorded conditions during day and night were 25.82 ± 3.79°C with 46.87 ± 4.76% RH and 22.29 ± 2.84°C with 49.29 ± 4.19% RH, respectively. The pots were bottom watered *via* separate plant saucers every second day with 50 ml tap water; and once a week with half-strength Hoagland nutrient solution (Hoagland and Arnon, 1950). The position of pots was rotated on a week basis to avoid spatial effects inside the greenhouse.

### *Rhizophagus irregularis* and *Trichoderma harzianum* root colonization

The presence *R. irregularis* in the roots of tomato plants was confirmed by incubating washed roots in 10% pre-heated KOH (≥85% p.a., ROTH, Karlsruhe, Germany) for 60 min. Subsequently, the fungal structures were stained with Trypan blue (ROTH) for 10 min at 80°C (Phillips and Hayman, 1970). Approximately 30 1-cm-long root segments were cut from each root sample to be observed under the microscope. The intensity of mycorrhizal structures was evaluated using a five-class system described by Trouvelot et al. (1986) under a binocular stereo microscope (Leica DM 4000 B LED). The quantification of mycorrhizal colonization in the root system (M%) was determined using the method of Mycocalc (https://www2.dijon.inra.fr/mychintec/Mycocalc-prg/download.html) (Supplementary Table S2). Similarly, the root samples of non-inoculated and *T. harzianum*-inoculated plants were also stained and microscopically observed, in order to confirm the absence of any mycorrhizal structures. Non-inoculated and *T. harzianum*-inoculated plants, in the roots of which mycorrhizal structures were observed, were excluded from analyses. The presence of *T. harzianum* in the soil samples of *T. harzianum*-inoculated plants was confirmed by using the plate count technique on PDA plates amended with 50 mg L^−1^ rose bengal (Applichem, Darmstadt, Germany) and 100 mg L^−1^ streptomycin sulphate (ROTH) (Martínez-Medina et al., 2009). The plates were incubated at 28°C in darkness, and colony forming units (CFUs) were counted 5 days later. Soil from non-inoculated and *R. irregularis*-inoculated plants was also sampled, in order to confirm the absence of *T. harzianum* (Supplementary Table S3). In case that *T. harzianum* CFUs had grown on PDA plates containing soil from non-inoculated and *R. irregularis*-inoculated plants, these plants were excluded from analyses.

### Y-tube olfactometer bioassays

To assess the olfactory response of *M. pygmaeus* toward the volatile blends emitted by differently root-inoculated plants in the presence and absence of *S. exigua* herbivory for 24 h, we performed a series of vertical Y-tube olfactometer assays (Takabayashi and Dicke, 1992). The Y-tube olfactometer (18 mm diameter, main arm 14 cm long, side arms 10 cm, 110° angle between the side arms) was connected to a pressurized air generation and flow system. Each side arm of the olfactometer was connected to a 1.5 L glass vessel containing one intact tomato plant. The air was generated by an air compressor (OLF2502, Jenpneumatic und Schlauchtechnik GmBh, Germany). The pressurized air was purified by passing through a glass bottle, humidified and entered into the odor chamber at a rate of 500 ± 50 ml/min regulated by a flowmeter. The airflow was measured with two flow sensors (PFMV510-1, SMC, Japan) adjusted next to each side arm of the Y-tube. Each pot was wrapped in aluminum foil to prevent the collection of volatiles emitted by soil or plastic. Plastic tubing was used for the connections between the different compartments of the set-up. To exclude visual cues and spatial effects, the bioassay took place in a darkroom. Additional lighting (LEICA KL1600 LED, the 4^th^ level) was provided at the end of each side arm of the Y-tube, and the odor chambers were screened off.

A single female predator (4^th^ to 5^th^ instar) was introduced into the main arm of the olfactometer and allowed to make a choice between the two arms (volatile sources). Each female was considered to have made a choice when walking at least 4 cm inside the chosen arm. Females that made no choice within 10 min were excluded from analysis. Each predator was used only once in the olfactometer bioassays and had no visual contact with the plants. At least 4 h before the bioassays, the predators were isolated from the colony and placed individually in a plastic container with perforated lid in a dark room for starvation. We recorded 40—50 replicates (individuals) depending on the treatment combination. After testing five insect individuals, the position of the two odor source chambers was switched between right and left side olfactometer arms in order to avoid positional effects. After every set of ten observations, the Y-tube and the odor chambers were thoroughly washed with ethanol 70% and allowed to dry in a drying oven, before being re-used. The bioassays were performed in a laboratory at 24 ± 2°C and 60%–70% RH from 10:30 to 18:00. Predator responses were assessed for all combinations of the following treatments: 1) non‐inoculated plants (Nm)‐ clean air, 2) non-inoculated plants (Nm)- non-inoculated *S. exigua*-infested plants (non-inoculated + h), 3) non-inoculated *S. exigua*-infested plants (Nm + h) - *R. irregularis*-inoculated *S. exigua*-infested plants (*Rhi* + h), 4) non-inoculated *S. exigua*-infested plants (Nm + h)- *T. harzianum*-inoculated *S. exigua*-infested plants (*Th* + h), 5) *R. irregularis*-inoculated *S. exigua*-infested plants (*Rhi* + h) - *T. harzianum*-inoculated *S. exigua*-infested plants (*Th* + h). All the tests were conducted in a random order to avoid any temporal effects.

### Impact of *Rhizophagus irregularis* and *Trichoderma harzianum* on the emission of *Spodoptera exigua* herbivory-induced volatiles

One day prior to herbivory bioassays, second- and third-instar *S. exigua* larvae were removed from the artificial diet and let to feed upon detached tomato leaves for acclimatization overnight. During acclimatization, the larvae were placed in the greenhouse next to the experimental chamber. The day after, three third-instar *S. exigua* larvae were placed on the apical leaflet of the third fully expanded leaf (counting from above) for 24 h. We used clip cages to confine the herbivores to one leaf. To control for potential effects of having a clip cage, the apical leaflet of the third fully expanded leaf of plants without herbivores was also enclosed into a clip cage for 24 h. After 24 h, the herbivores were removed and the volatile collection followed.

### Volatile collection and analysis

To investigate the effect of beneficial microbes on the emission of herbivore-induced volatiles, we collected the volatiles emitted by both herbivore- and non-herbivore-infested plants in the greenhouse. Volatiles were collected from Nm (non-inoculated, *n* = 6), Nm + h (non-inoculated + *S. exigua*, *n* = 7), *Rhi* (*R. irregularis*-inoculated, *n* = 7), *Rhi* + h (*R. irregularis* + *S. exigua*, *n* = 7), *Th* (*T. harzianum*-inoculated, *n* = 7) and *Th* + h (*T. harzianum* + *S. exigua*, *n* = 7) plants. The collection of volatiles took place from 10:00 to 14:00; the temperature was 25.61 ± 1.19 °C and 42.27 ± 2.09% RH. We followed the passive trapping method described by Kallenbach et al. (2015) using polydimethysiloxane (PDMS) tubes. Metallic wire cuttings were mounted on the clip cage adjusted at every experimental plant. Two PDMS tubes were used as technical replicates in each clip cage. After 24 h of *S. exigua* herbivory, the tubes were left to collect volatiles for 4 h. The background air volatiles (blank samples) were also collected using the same set-up at each corner of the greenhouse in the absence of plants. When the passive trapping was completed, each pair of PDMS tubes was stored into individually labelled 4 ml glass vials, tightly closed with a screw cap and immediately kept at -20°C until analyzed.

Subsequently, one technical replicate from each PDMS tube pair that was used to collect volatiles was transferred into an empty stainless-steel tube (MARKES, Llantrisant, UK), and the samples were analyzed using a Thermal Desorption- Gas Chromatograph—Mass Spectrometer (TD-GC-MS). TD-GC-MS consisted of a thermodesorption unit (MARKES, Unity 2, Llantrisant, UK), equipped with an autosampler (MARKES, Ultra 50/50), and a gas chromatograph (Bruker, GC-456, Bremen, Germany) connected to a triple-quad mass spectrometer (Bruker, SCION, Hamburg, Germany). The tubes were desorbed following the conditions described below: Dry purge 5 min at 20 ml/min, pre-purge 1 min (or 2 min) at 10 ml/min to remove remaining water, desorption 8 min at 200°C with 60 ml/min, trap temperature 0°C, pre-trap fire purge 1 min at 60 ml/min, split flow 20 ml/min, trap heated to 230°C at maximum rate and hold for 4 min. The separation of compounds took place on a DB-WAX column (30 m × 0.25 mm inner diameter x 0.25 μm film thickness, manufactured by Restek (Bellefonte, PA, United States, distributed by Analytik, Bad Homburg, Germany). The conditions for GC were set as following: 60°C [hold 1 min 30°C/min to 150°C, 10°C/min to 200°C and 30°C/min to 230°C (hold 5 min)]. Helium was used a carrier gas with a constant flow of 1 ml/min. The mass spectrometry conditions were set at 40°C for the manifold, 240°C at the transfer line, and 220°C for the ion source. The scan range was 33–500 m/z for a full scan, and the scan time was 250 ms (Sam et al., 2021).

A large body of scientific studies have showed that the volatile blends emitted by tomato plants after herbivory include compounds belonging to the chemical classes of green leaf volatiles (GLVs), terpenoids and the aromatic compound methyl salicylate (MeSA) (Ament et al., 2004; Sasso et al., 2007; Battaglia et al., 2013; Coppola et al., 2017; Pappas et al., 2018). Therefore, we followed a targeted volatile analysis approach to annotate compounds belonging to these chemical classes. For our analysis, we selected the most prominent peaks in the chromatograms (signal to noise ratio >10), that could be assigned to GLVs, monoterpenes and sesquiterpenes and methyl salicylate. That resulted in 18 peaks. The compounds were annotated with spectral libraries (National Institute of Standards and Technology (NIST 20), Wiley 20) and compared to retention indices from the literature. When possible, peaks were additionally identified by injection of authentic standards.

### Extraction of tomato leaf total RNA and cDNA synthesis

After completing the collection of volatile compounds, the leaflets enclosed into the clip cages from both *S. exigua*-infested and non-infested plants of all treatments were harvested, flash-frozen in liquid nitrogen, according to Bandoly and Steppuhn (2016), and stored at −80°C for further analyses. To explore the effects of root symbionts on indirect defense responses of tomato in response to *S. exigua* herbivory, we measured the expression levels of two jasmonic acid (JA) pathway-related genes: the lipoxygenases *LOX* and *LOXA*, one allene oxide synthase 2 (*AOS2*) and one terpene synthase gene (*TPS5*). *LOX*, *LOXA* and *AOS2*, which are components of octadecanoid signal transduction pathway, are involved in the biosynthesis of JA and green leaf volatiles (GLVs); and *TPS5* contributes in the biosynthesis of the monoterpene linalool (Cao et al., 2014). Among the SA-pathway-related genes, we selected the genes phenylalanine ammonia-lyase (*PAL*) and the salicylic acid carboxyl methyltransferase (*SAMT*). In the phenylpropanoids pathway, *PAL* is involved into the salicylic acid (SA) biosynthesis and *SAMT* modifies SA into methyl-salicylate (MeSA), which is volatile (Ament et al., 2004). RNA was extracted from leaf tissues according to Oñate-Sánchez and Vicente-Carbajosa (2008) with slight modifications. The samples were treated with DNAse Ⅰ (Thermo Scientific) and cDNA was synthesized with a 1st-strand cDNA synthesis kit (Thermo Scientific) as described in the protocol. Quantitative PCR was conducted using the SYBR^®^ Green Supermix (Bio-RAD, Germany) following the manufacturer’s instructions and a thermal cycler (Bio-RAD, C1000 Touch Thermal Cycler CFX 384, Germany) with the following cycling program: 2 min 50°C and 10 min 95°C, 40 cycles of 15 sec 95°C and 1 min 60°C, followed by a melting curve analysis. The sequences of the gene-specific primers used in qPCR are shown in Supplementary Table S9. The 1^st^ strand cDNA synthesized was normalized based on the expression of the housekeeping gene *Solanum lycopersicum* elongation factor 1a (*SlEF1a*). The chosen housekeeping gene was stable among the samples processed and within the analysis conditions. In total, six to nine biological replicates of tomato RNA samples were analyzed with three technical replicates for each gene (Supplementary Table S10). The relative gene expression level (2-∆∆C_t_) was calculated on Microsoft Excel according to Pfaffl (2001).

### Statistical analysis

For the Y-tube olfactometer assays, the null hypothesis that the females of *M. pygmaeus* showed no preference for either arm of the olfactometer was tested using χ^2^ test in R (Team 2013). Differences in the peak intensities measured for the targeted volatile compounds and the expression levels of the selected genes were analyzed in R (Team 2013). Two-way analysis of variance (ANOVA) was used to determine the effect of the root microbes used to inoculate the roots of the plants, the effect of herbivory and the combined effect of the two afore-mentioned factors. Tukey multiple comparisons of means followed ANOVA to identify significant differences among the treatments. Prior to two-way ANOVA, all the data were log-transformed. Levene’s test was used to test the homogeneity of variance across groups, and Shapiro test was used to check for the normal distribution of residuals. When the assumptions for two-way ANOVA were not met, one-way ANOVA was conducted only for the samples collected from herbivore-infested plants, followed by Tukey post hoc tests. Prior to one-way ANOVA, all the data were log-transformed. In the cases, where the assumptions for one-way ANOVA were not met, Kruskal Wallis test was performed for the samples collected from herbivore-infested plants, followed by Dunn’s multiple comparisons test. Boxplots were plotted in R using the package ggplot2 (Wickham, 2016). Principal Component Analysis (PCA) was conducted in R (Team 2013) to explain the variation in the volatile peak intensity data.

## Results

### *Macrolophus pygmaeus* choice

By using a Y-tube olfactometer, we investigated whether the inoculation of tomato roots with beneficial microbes could affect the attraction of the omnivorous predator *M. pygmaeus* towards *S. exigua*-infested plants. Therefore, we set up a Y-tube olfactometer assay, where *M. pygmaeus* females were allowed to make a choice between the following options: 1) non-inoculated tomato plants versus clean air (Nm *vs.* air), 2) non-inoculated versus non-inoculated *S. exigua*-infested plants (Nm *vs.* Nm + h), 3) non-inoculated *S. exigua*-infested versus *R. irregularis*-inoculated *S. exigua*-infested plants (Nm + h *vs. Rhi* + h), 4) non-inoculated *S. exigua*-infested versus *T. harzianum*-inoculated *S. exigua*-infested plants (Nm + h *vs. Th* + h) and 5) *R. irregularis*-inoculated *S. exigua*-infested versus *T. harzianum*-inoculated *S. exigua*-infested plants (*Rhi* + h *vs. Th* + h).

**FIGURE 1:**
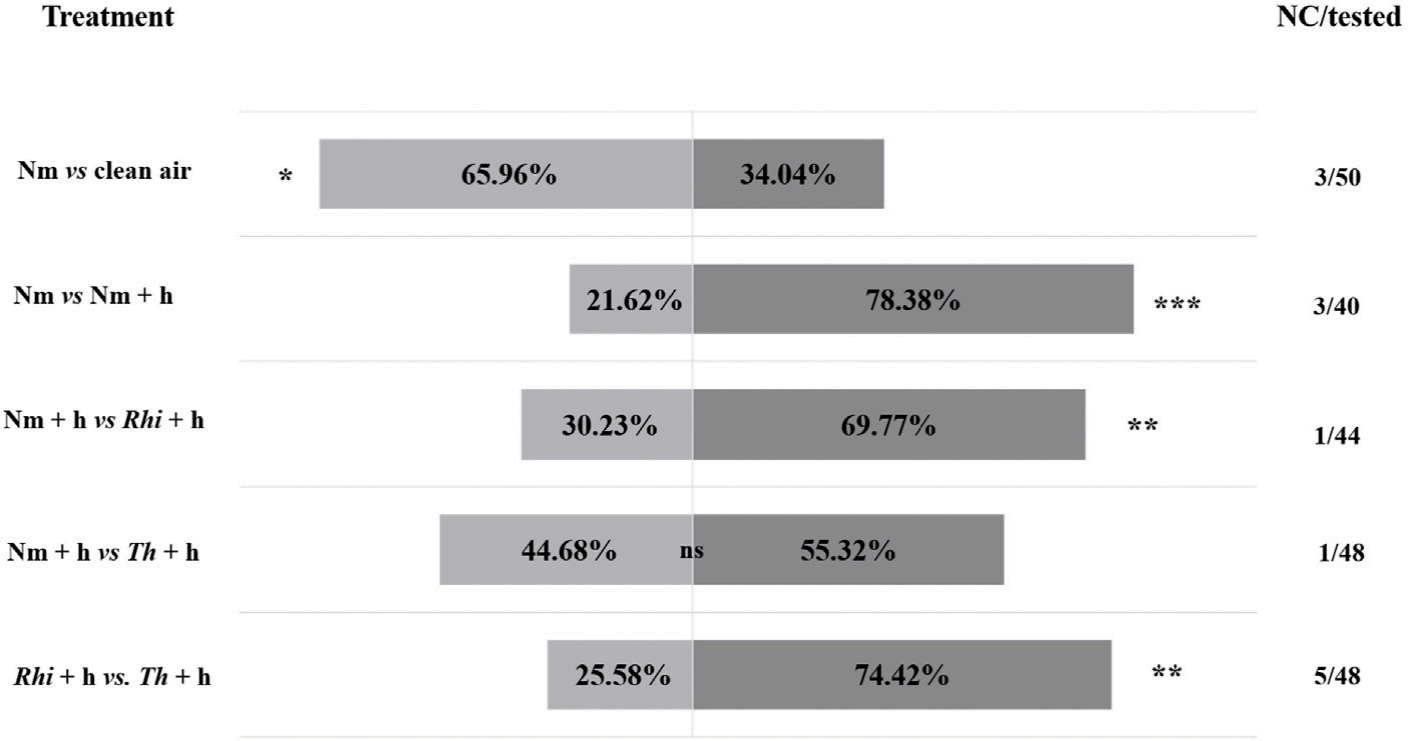
Responses of *Macrolophus pygmaeus* female predators to the volatiles emitted by non-inoculated (Nm), non-inoculated + *S. exigua* (Nm + h), *Rhizophagus irregularis*-inoculated + *S. exigua* (*Rhi +* h), *Trichoderma harzianum*-inoculated + *S. exigua* (*Th +* h) plants or clean air (air). At the sides of each bar, are shown the percentages of the predator females that moved towards the corresponding odor source based on the comparison indicated on the left side of the bars. The no choice/tested column on the right of each bar indicates the number of female predators that made no choice over the total number of tested predators (44–50 predators depending on the treatment combination were tested). In total, 13 out of 230 predators failed to make a choice. Asterisks indicate significant differences after performing a χ^2^ test: ns (*p* > 0.05), ∗ (*p* < 0.05), ∗∗ (*p* < 0.01), and ∗∗∗ (*p* < 0.001).

Our results showed that *M. pygmaeus* females preferred Nm tomato plants to clean air (Figure 1, *χ* ^*2*^ (1, *n* = 47) = 4.79; *p* = 0.03). In addition, the females were strongly attracted by the volatiles emitted by Nm + h infested plants over the Nm ones (Figure 1, *χ* ^*2*^ (1, *n* = 37) = 11.92; *p* = 0.0006). The comparison between Nm + h and *Rhi* + h plants, showed a stronger preference to the *Rhi* + h ones (Figure 1, *χ* ^*2*^ (1, *n* = 43) = 6.72; *p* = 0.001). The volatiles emitted by *Th* + h plants, attracted more female predators than the volatiles emitted by Nm + h plants. However, the difference between the two was not statistically significant (Figure 1, *χ* ^*2*^ (1, *n* = 47) = 0.53; *p* = 0.47). The comparison between *Rhi* and *Th* plants both under herbivory showed that the female predators were stronger attracted by the *Th* + h over the *Rhi + h* plants (Figure 1, χ^*2*^ (2, *n* = 43) = 10.3; *p* = 0.01).

### Tomato volatiles

In order to investigate the compounds that might be responsible for affecting the choices of *M. pygmaeus* females recorded in the Y-tube olfactometer assays, we collected volatiles from non-inoculated and root-inoculated plants with and without *S. exigua* larvae infestation (Nm, Nm + h *Rhi*, *Rhi* + h, *Th*, *Th* + h). Targeted analysis of our volatile data resulted in a list of 18 compounds that were further analyzed. Among them, we (tentatively) identified methyl salicylate, compounds belonging to the distinct classes of C6 green leaf volatiles (GLVs), monoterpenes and sesquiterpenes.

**FIGURE 2:**
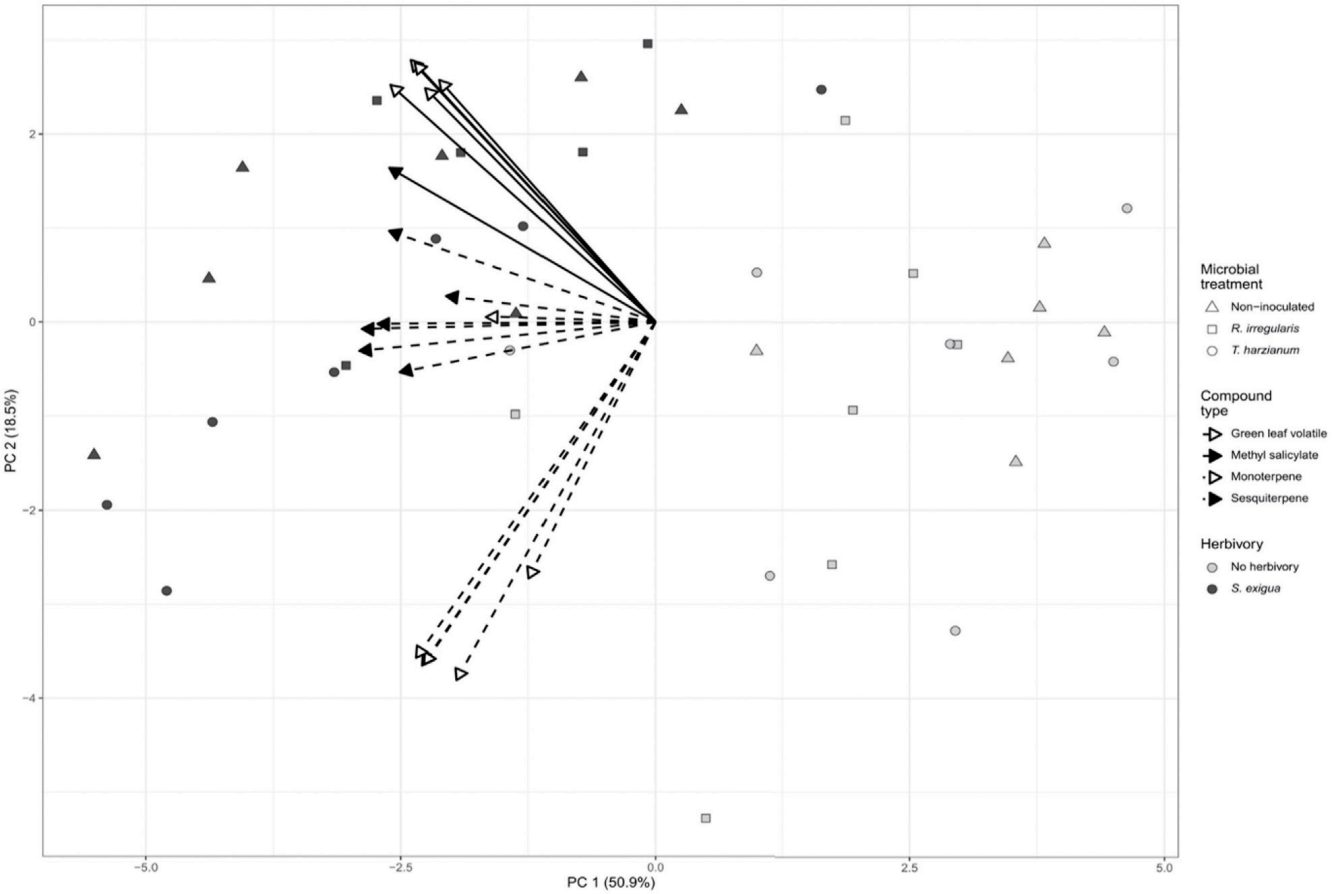
Principal component analysis (PCA) biplot describing the volatile emissions of non-inoculated (Nm), *Rhizophagus irregularis*-inoculated (*Rhi*), *Trichoderma harzianum*-inoculated (*Th*) tomato plants non-infested or infested by *Spodoptera exigua* larvae for 24 h. Principal component analysis (PCA) was performed for 18 selected volatile compounds. Symbols represent the biological replicates within each microbial treatment: Nm (triangle); *Rhi* (square) *Th* (circle). Filled symbols represent *S. exigua*-infested plants, whereas not filled symbols represent non-infested plants. The arrows point to the volatile organic compound classes that contributed to the separation of the samples based on our targeted analysis. Continuous line arrows without a filled head point to the green leaf volatiles; with a filled head point to methyl salicylate. Perforated line arrows without a filled head point to monoterpenes; with a filled head point to the sesquiterpenes.

Principal component analysis (PCA) revealed that the volatile emissions of herbivore-infested plants differed from the volatile emissions of non-herbivore-infested plants. As shown by PCA, the first two principal components explained 69.4% of combined variance and separated our samples between herbivore- and non-herbivore-infested (Figure 2). *Spodoptera exigua* herbivory upon the leaves of tomato is considered the factor driving the separation among our samples.

**FIGURE 3:**
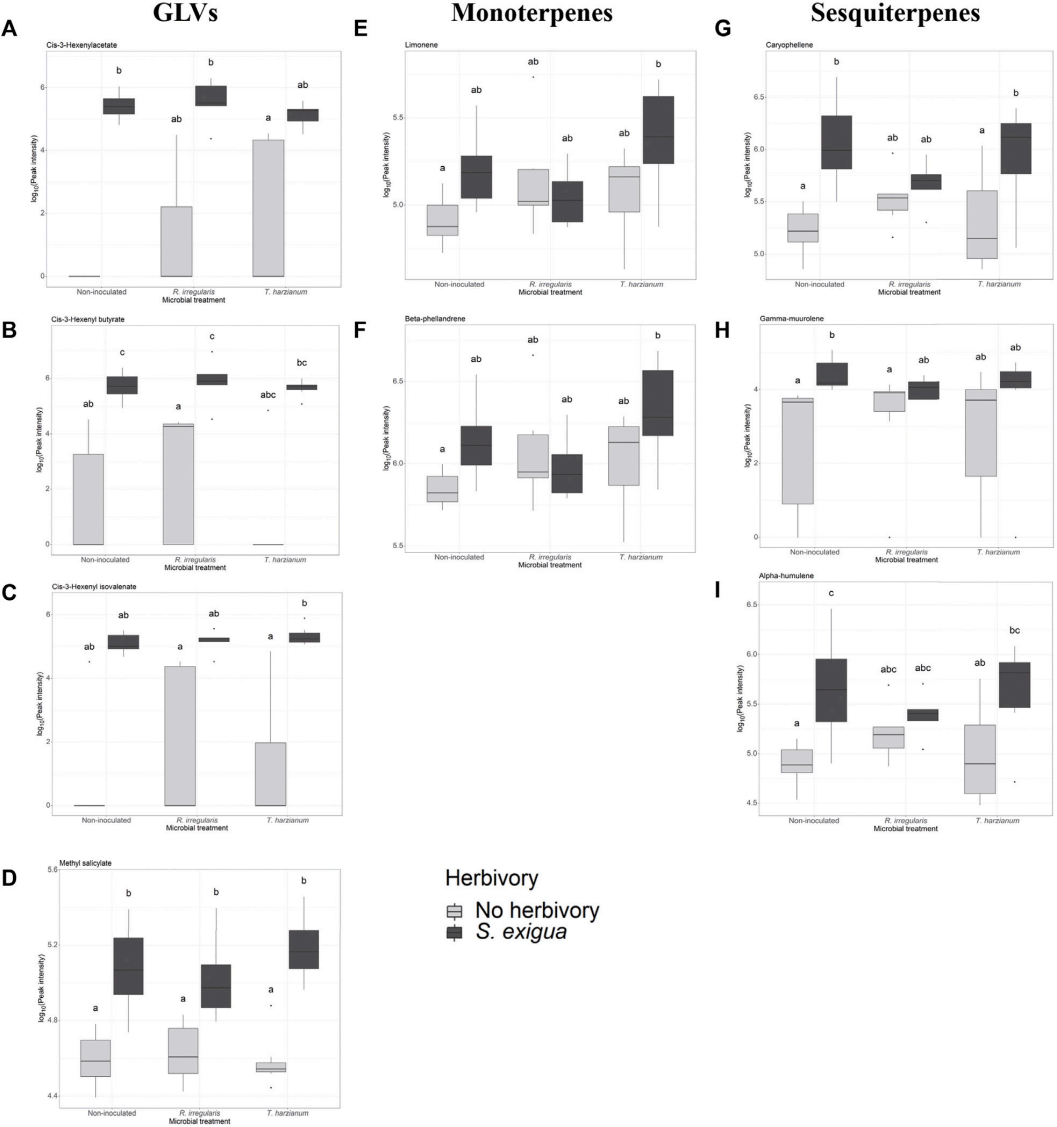
Selection of volatile compounds emitted by non-inoculated and root-inoculated tomato plants non-infested or infested by *Spodoptera exigua* larvae for 24 h. The compounds depicted belong to the class of green leaf volatiles **(A–C)**, methyl salicylate **(D)**, monoterpenes **(E,F)**, and sesquiterpenes **(G–I)**. The compounds shown were selected based on the effect of *S. exigua* herbivory on their emission. Treatments are: control plants symbolized as Non-inoculated, *Rhizophagus irregularis*-root inoculated plants symbolized as *R. irregularis* and *Trichoderma harzianum* root-inoculated plants symbolized as *T. harzianum*. Light gray-colored boxplots represent non-herbivore-infested plants and dark gray-colored boxplots represent *S. exigua*-infested plants. Significant differences between treatments are indicated by different letters after Tukey’s *post hoc* tests after two-way ANOVA: *p* < 0.05.

Next, we investigated whether herbivory of *S. exigua* upon tomato leaves, tomato root inoculation with beneficial microbes and the interaction effect between the two aforementioned factors influenced the induction of the targeted volatile compounds. For the GLVs, *S. exigua* herbivory demonstrated a predominant effect on the emission of three compounds. In particular, this was observed for the green leaf volatile cis-3-hexenylacetate (*p*_h_ = 0.002, Table 1; Supplementary Table S2; Figure 3A), the compound identified as cis-3-hexenylbutyrate (*p*_h_ = 2.06e-05, Table 1; Supplementary Table S2; Figure 3B) and the compound tentatively identified as cis-3 hexenyl isovalerate (*p*_h_ = 0.0001, Table 1; Supplementary Table S2; Figure 3C). For the other two GLVs [unknown GLV 1 and the compound tentatively identified as 3-hexen-1-ol, propanoate, (*Z*)-] in our analysis, two-way ANOVA showed that none of the factors tested influenced the release of these compounds from tomato plants in response to larval infestation (Table 1; Supplementary Table S2; Supplementary Figure S1A,B, respectively). The interaction effect between herbivory and beneficial root microbes was not shown as significant for any of the five GLVs found. Similarly, root inoculation of our experimental plants with beneficial microbes prior to herbivory did not affect the synthesis and emission of any of the green leaf volatiles we focused on. Herbivory by *S. exigua* larvae significantly affected the emission of the volatile methyl salicylate (MeSA) (*p*_h_ = 9.2e-10, Table 1; Supplementary Table S2; Figure 3D) as well. Both the effect of root symbionts and the interaction effect between herbivory and the root symbionts used were not significant in shaping MeSA emissions.

**TABLE 1:** Volatiles emitted by tomato plants according to their measured retention time and calculated Kovats retention index (RI).

Volatile	Calculated kovats RI	Two-way ANOVA		
		Microbe (m)	Interaction (h x m)	Herbivore (h)
cis-3-hexenyl acetate^a^	1318	0.101	0.484	0.002 (**)
unknown GLV1	1379	0.631	0.105	0.271
3 hexen-1-ol, propanoate (*Z*)-^b^	1384	0.672	NA	0.99
cis-3-hexenyl butyrate^a^	1462	0.349	0.551	2.06e-05 (***)
cis-3-hexenyl isovalerate^c^	1473	0.331	0.76	0.0001 (***)
methyl salicylate	1790	0.476	0.369	9.2e-10 (***)
unknown monoterpene 1	1141	0.127	0.368	0.144
limonene^a^	1207	0.186	0.071 (.)	0.023 (*)
*β*-phellandrene^d^	1219	0.189	0.106	0.036 (*)
trans-*β*-ocimene^a^	1254	0.574	0.349	0.025 (*)
unknown sesquiterpene 1	1595	0.273	0.8	0.88
caryophellene^a^	1621	0.818	0.071 (.)	4.72e-05 (***)
unknown sesquiterpene 2	1654	0.086 (.)	0.274	0.312
*γ*-muurolene^d^	1685	0.159	0.346	0.001 (**)
*α*-humulene^d^	1690	0.983	0.2	0.0002 (***)

^a^ Authentic standard.

^b^ Werkhoff et al. (1998).

^c^ Kawakami et al. (1995).

^d^ Lopes et al. (2004).

Compounds were identified by comparison to an authentic standard^a^ or tentatively identified by comparison to RI values in the literature^b,c,d^, when possible. In this table, the volatile compounds of which the *p*-values have been calculated with two-way ANOVA are presented with *Microbe* (m), *Herbivore* (h) and their interaction (m x h) as factors. Asterisks indicate significant differences after performing two-way ANOVA: ∗ (*p* < 0.05), ∗∗ (*p* < 0.01), ∗∗∗ (*p* < 0.001).

In our volatile analysis, we also targeted six monoterpenes. The effect of root symbionts was marginally significant for the emission of the monoterpene tentatively identified as *α*-phellandrene (*p*_m_ = 0.08, Table 2; Supplementary Table S3; Supplementary Figure S2B) and the monoterpene tentatively identified as *α*-terpinene (*p*_m_ = 0.06, Table 2; Supplementary Table S4; Supplementary Figure S2A). As the post hoc tests showed, there was a marginally significant difference in the emission of the monoterpene *α*-phellandrene between the *T. harzianum*- and *R. irregularis*-inoculated plants (*p*_adj_ = 0.08, Supplementary Table S3). Similarly, a significant difference was found between the non-inoculated and mycorrhizal plants under herbivory in the release of the monoterpene *α*-terpinene (*p*_adj_ = 0.05, Supplementary Table S4). On the other hand, *S. exigua* herbivory was the main factor driving the emission of the monoterpene identified as limonene (*p*_h_ = 0.02, Table 1; Supplementary Table S2; Figure 3E). Interestingly, for the same volatile compound, the interaction effect between herbivory and the root symbionts used was marginally significant (*p*_m x h_ = 0.07, Table 1; Supplementary Table S2). Conversely, the emission of the monoterpene tentatively identified as *β*-phellandrene was significantly affected exclusively by *S. exigua* herbivory at *p*_h_ = 0.03 (Table 1; Supplementary Table S2; Figure 3F). Herbivory had the same significant effect on the emission of the monoterpene identified as trans-*β*-ocimene and *p*_h_ = 0.02, (Table 1; Supplementary Table S2; Supplementary Figure S1D).

**TABLE 2:** Volatiles emitted by tomato plants according to their measured retention time and calculated Kovats retention index (RI).

Volatile compound	Calculated kovats RI	One-way ANOVA	Kruskal–Wallis test
		microbe (m)	microbe (m)
*α*-phellandrene^a^	1175	0.086 (.)	-
*α*-terpinene^a^	1187	-	0.065 (.)
unknown sesquiterpene 3	1707	-	0.858

^a^ Lopes et al. (2004).

Compounds were identified by comparison to an authentic standard^a^ or tentatively identified by comparison to RI values in the literature^b,c,d^, when possible. In this table, the volatile compounds of which the *p*-values have been calculated with one-way ANOVA or Kruskal–Wallis test among herbivore-infested samples are presented with *Microbe* (m) as factor. Asterisks indicate significant differences after performing one-way ANOVA or Kruskal–Wallis test: ∗ (*p* < 0.05), ∗∗ (*p* < 0.01), ∗∗∗ (*p* < 0.001).

Apart from monoterpenes, in our analysis, we also focused on six sesquiterpenes. Herbivory strongly affected the levels of caryophellene (*p*_h_ = 4.72e -05, Table 1; Supplementary Table S2; Figure 3G). In addition, a weak interactive effect between *S. exigua* herbivory and the root symbionts was found for the same compound (*p*_h x m_ = 0.07, Table 1; Supplementary Table S2). Similarly, the beneficial microbes used to inoculate the plants were shown to marginally influence the emission levels of the unknown sesquiterpene 2 (*p*_m_ = 0.08, Table 1; Supplementary Table S2; Supplementary Figure S1F). Lastly, for the sesquiterpenes tentatively identified as *γ*-muurolene and *α*-humulene, larval herbivory was shown as the main factor influencing their emission with *p*_h_ = 0.001 and *p*_h_ = 0.0002, respectively (Table 1; Supplementary Table S2; Figure 3H,I, respectively).

### Tomato indirect defense gene expression

**FIGURE 4:**
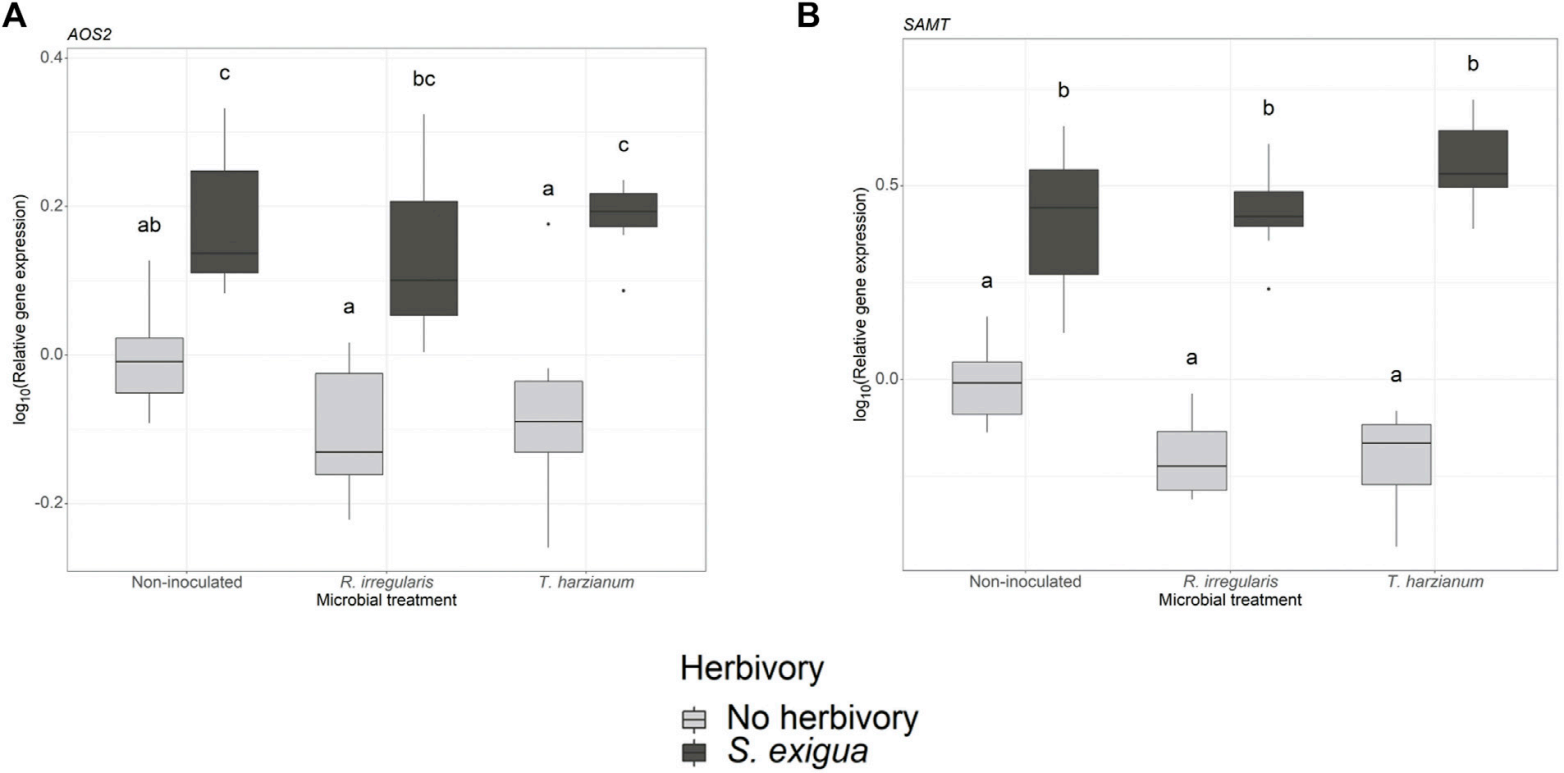
Transcript levels of two defense-related genes in non-inoculated and root-inoculated tomato plants non-infested or infested by *Spodoptera exigua* larvae for 24 h. Panel **(A)** depicts the transcript levels of the gene allene oxide synthase 2 (AOS2), and panel **(B)** depicts the transcript levels of the gene salicylic acid methyl transferase (SAMT). The genes depicted were selected based on the effect of *S. exigua* herbivory, or beneficial microbes on their transcript levels. Values are the average of the three technical replicates for each of six-seven biological replicates per treatment. Expression levels for the selected genes were normalized to the mean of tomato elongation factor 1a gene expression levels in each sample as reference. Light gray-colored boxplots represent non-herbivore-infested plants and dark gray-colored boxplots represent *S. exigua*-infested plants. Significant differences between treatments are indicated by different letters after Tukey’s *post hoc* tests after two-way ANOVA: *p* < 0.05.

In our gene expression analysis, we focused on selected genes, which are involved in the jasmonic acid, salicylic acid and terpenoid biosynthetic pathways. For the jasmonic acid pathway-related genes, we observed that the transcriptional levels of the lipoxygenases *LOXA* and *LOX* were not significantly affected by the root symbionts (one-way ANOVA, *p*_m_ = 0.58 (Supplementary Table S7; Supplementary Figure S3B) and *p*_m_ = 0.5 (Supplementary Table S7; Supplementary Figure S3A) for *LOXA* and *LOX*, respectively). Conversely, herbivory was the main factor driving the expression levels of the gene *allene oxide synthase 2* (*AOS2*; *p*_h_ = 2.389e-10 (Supplementary Table S5, S6; Figure 4A). However, neither the beneficial root microbes nor the interactive effect of herbivory and root symbionts significantly affected *AOS2* gene expression.

As a representative of terpenoid biosynthesis, we investigated the expression levels of the terpene synthase gene, *TPS5*. As it was shown, among the herbivore-infested samples, the root symbionts had no significant effect in determining the *TPS5* gene expression levels (*p*_m_ = 0.29, Supplementary Table S8; Supplementary Figure S3D).

We also measured the transcript levels of two genes involved in the salicylic acid (SA) biosynthetic pathway. At first, one-way ANOVA among the samples collected from larvae-infested plants, showed that arbuscular mycorrhiza and *T. harzianum* did not affect the expression of the gene phenylalanine ammonia-lyase (*PAL*) (*p*_m_ = 0.45, Supplementary Table S7; Supplementary Figure S3C). In the same pathway, we also measured the transcript levels of the gene salicylic acid methyl transferase (*SAMT*). Two-way ANOVA showed that herbivory strongly influenced the expression of *SAMT* (*p*_h_ < 2.2e-16, Supplementary Table S5, S6; Figure 4B). Interestingly, the interaction effect between *S. exigua* infestation and the root symbionts also played a significant role in modulating the expression levels of this gene (*p*_h x m_ = 0.004, Supplementary Table S5, S6).

## Discussion

In our study, we found that tomato plants damaged by *Spodoptera exigua* caterpillars were highly attractive to *Macrolophus pygmaeus* females. Among herbivore infested plants, plants inoculated with beneficial microbes were more attractive, in particularly those infested with the mycorrhizal fungus *Rhizophagus*. However, when given the choice among the two, *M. pygmaeus* preferred herbivore- induced plants inoculated with *Trichoderma*. Comparative volatile and gene transcription analysis of herbivore-induced plants with and without beneficial microbes, however, did not yield a clear mechanistic basis for the observed choice patterns. The only exception to this might be the interactive effect of caterpillar and beneficial microbes on *SAMT*, the gene responsible for MeSA production. MeSA is well-known as a volatile cue attracting predators, including *Macrolophus* (Silva et al., 2021).

However, we found a strong effect of herbivore feeding on predator response, which we could clearly link to volatile emissions. Among non-inoculated plants, female predators were stronger attracted by the herbivore-damaged over undamaged plants. As shown in our volatile analysis, *S. exigua* larval herbivory resulted in the enhanced emission of a blend of volatiles including GLVs, such as cis-3-hexenyl acetate, methyl salicylate, and several terpenoids, such as *β*-phellandrene, trans-*β*-ocimene, caryophellene and *α*-humulene. Therefore, these volatile compounds may have affected the attraction of the female predators towards herbivore-damaged plants. In accordance with our findings, studies have shown that the GLVs (*Z*)-3-hexen-1-ol and (*Z*)-hexenyl acetate, and the volatile methyl salicylate determine the attraction of several parasitoid and predator species including *M. pygmaeus* (James, 2003a; b;Moayeri et al., 2007; Uefune et al., 2011; Salamanca et al., 2017; Silva et al., 2021). Moreover, the terpenoids *β*-phellandrene, (*E*)-ocimene, (*Z*)-ocimene, *β*-caryophellene and *α*-humulene have also been shown to attract the predator *M. pygmaeus* (De Backer et al., 2015). The strong effect of *S. exigua* herbivory was also depicted by the upregulation of *AOS2* and *SAMT,* genes involved in the oxylipin and phenylpropanoid pathways, respectively. These two pathways play a major role in HIPV biosynthesis. Notably, in the case of *SAMT,* an interaction effect between herbivory and beneficial microbes was also detected, underlining the ability of root symbionts to enhance indirect defense in response to herbivory and increase the attraction of natural enemies (Schausberger et al., 2012; Battaglia et al., 2013).

Among herbivore-infested plants, female predators showed significantly higher attraction towards *R. irregularis-*inoculated plants compared to the non-inoculated ones. Several studies have shown that beneficial root microbes, such as mycorrhizal fungi, are capable of modulating plant defense responses through the activation of induced systemic resistance or priming (Pineda et al., 2010; Martínez-Medina et al., 2017; Rasmann et al., 2017; Shikano et al., 2017). Our volatile analysis showed that mycorrhization only weakly affected the emission of a monoterpene, tentatively identified as *α*-terpinene. Notably, *α*-terpinene has been reported as one of the most influencing compounds on determining the attraction of *M. pygmaeus* predators towards *T. absoluta*-infested tomato plants (De Backer et al., 2015). The study of Prieto et al. (2017) reported improved foraging behavior and life history traits of *M. pygmaeus* on tomato plants root-inoculated with the isolate BEG 72 of *R. irregularis*. In particular, the authors showed that mycorrhizal plants attracted both female and male predators more strongly, were highly preferred for oviposition by reproductive females, and subsequently hosted a higher number of newborn nymphs (Prieto et al., 2017). Even if the object of the aforementioned study was not the investigation of the volatiles emitted by mycorrhizal and non-mycorrhizal tomato plants, the authors hypothesized that quantitative and/or qualitative changes in the blend of VOCs released might have mediated the higher attraction of the predator towards the leaves of mycorrhizal plants. We thus hypothesize that the volatile *α*-terpinene might have played an important role in the stronger attraction of *M. pygmaeus* towards mycorrhizal herbivore-infested plants in our study. Our finding combined with the results of Prieto et al. (2017) indicates that root colonization by *R. irregularis* could facilitate the establishment of the *Macrolophus* colonies in tomato crops in fields or greenhouses, provided there is abundance of prey, thus resulting in an effective and more sustainable way to protect the crops against herbivores.

Our results showed that root inoculation with beneficial fungi did not alter the expression levels of the *TPS5* gene in response to caterpillar herbivory. In our study, we investigated the expression levels of the gene coding for *TPS5*, which has been determined as a linalool synthase (Cao et al., 2014). The synthesis of *α*-terpinene in tomato is catalyzed by the enzymes *TPS9* and *TPS20* (Zhou and Pichersky, 2020), which might be the reason we did not observe any effect of mycorrhization on the expression levels of the terpene synthase gene chosen to be tested.

Several studies have reported significantly higher attraction of parasitoids and predators towards *Trichoderma*-inoculated tomato plants (Battaglia et al., 2013; Coppola et al., 2017). However, in our Y-tube experiments, the female predators did not prefer *T. harzianum-*inoculated to non-inoculated plants. A reason for this difference might be the different fungal strains, insect herbivores and/or tomato plant cultivars used in various studies.

Considering the above, it was all the more interesting that herbivore-induced plants with *T. harzianum* were significantly more attractive to predator females than the mycorrhizal plants. Our volatile analysis showed there was a marginally significant difference between *T. harzianum*-inoculated and mycorrhizal plants regarding the emission of a monoterpene, tentatively identified as *α*-phellandrene. This is in line with other studies, reporting that *Trichoderma spp*. can alter the concentration of terpenoids emitted by host plants in response to herbivory (Battaglia et al., 2013; Contreras-Cornejo et al., 2018a; Contreras-Cornejo et al., 2018b). Alpha-phellandrene was annotated among the volatile compounds emitted by *T. longibrachiatum* MK1-inoculated tomato plants, even if not significant (Battaglia et al., 2013). The same plants exhibited promoted plant development and were significantly more attractive for *M. pygmaeus* compared to the non-colonized ones (Battaglia et al., 2013). The difference observed in the degree of significance of *α*-phellandrene between our study and the study of Battaglia et al. (2013) might be attributed to the difference between the *Trichoderma* strains used. We thus hypothesize that *α*-phellandrene might be involved in the mechanism used by *Trichoderma* strains to induce indirect defense responses against herbivores in tomato plants. In this frame, sustainable crop protection strategies that employ *M. pygmaeus* zoophytophagous predators and *Trichoderma* fungal strains could work synergistically in protecting tomato crops against herbivores.

To our knowledge, this is the first study so far, to compare the responses of female predators towards mycorrhizal and *Trichoderma*-inoculated tomato plants under herbivory. Arbuscular mycorrhizal fungi and *Trichoderma* species use different mechanisms of colonization and induction of biochemical, physiological and molecular responses on the host plans (Strack et al., 2003; Smith and Smith, 2011; Hermosa et al., 2012). Therefore, we hypothesize that the presence of *T. harzianum* on tomato roots might led to slightly increased emission of *α*-phellandrene compared to the mycorrhizal plants. This difference in the volatile blend of *T. harzianum* plants might subsequently influenced the responses of *M. pygmaeus* female predators. Overall, both *α*-phellandrene and *α*-terpinene showed higher emissions in the microbe-inoculated plants, thus we assume that the two compounds may have influenced the attraction of the predator. *Macrolophus pygmaeus* predators are broadly released in greenhouses to protect tomato crops against herbivores (Castañé et al., 2004; Urbaneja et al., 2012). Therefore, choice experiments to compare the responses of female predators to the volatile compounds *α*-phellandrene and *α*-terpinene are required to elucidate which of the two compounds might be more effective.

## Conclusion

Collectively, our results show that, despite the dominant effect of herbivory on the synthesis and emission of volatiles; beneficial microbes also show potential of altering the biosynthesis and release of these compounds. Our findings combined with the results of other studies in the field pinpoint the role that root symbionts could play in the application of integrated pest management practices and sustainable agriculture. To this direction, more studies investigating the ability of different beneficial microbes to modulate indirect defenses in response to herbivory through the attraction of natural enemies of the insect herbivores are required.

## Data Availability

The datasets presented in this study can be found in online repositories. The names of the repository/repositories and accession number(s) can be found below: https://www.zenodo.org/, https://doi.org/10.5281/zenodo.6698584.
