# Supplementary figures and images for: Root symbionts alter herbivore-induced indirect defenses of tomato plants by enhancing predator attraction
Source: Front Physiol. 2022 Oct 21;13:1003746. doi: 10.3389/fphys.2022.1003746 (PMC9634184; doi:10.3389/fphys.2022.1003746)

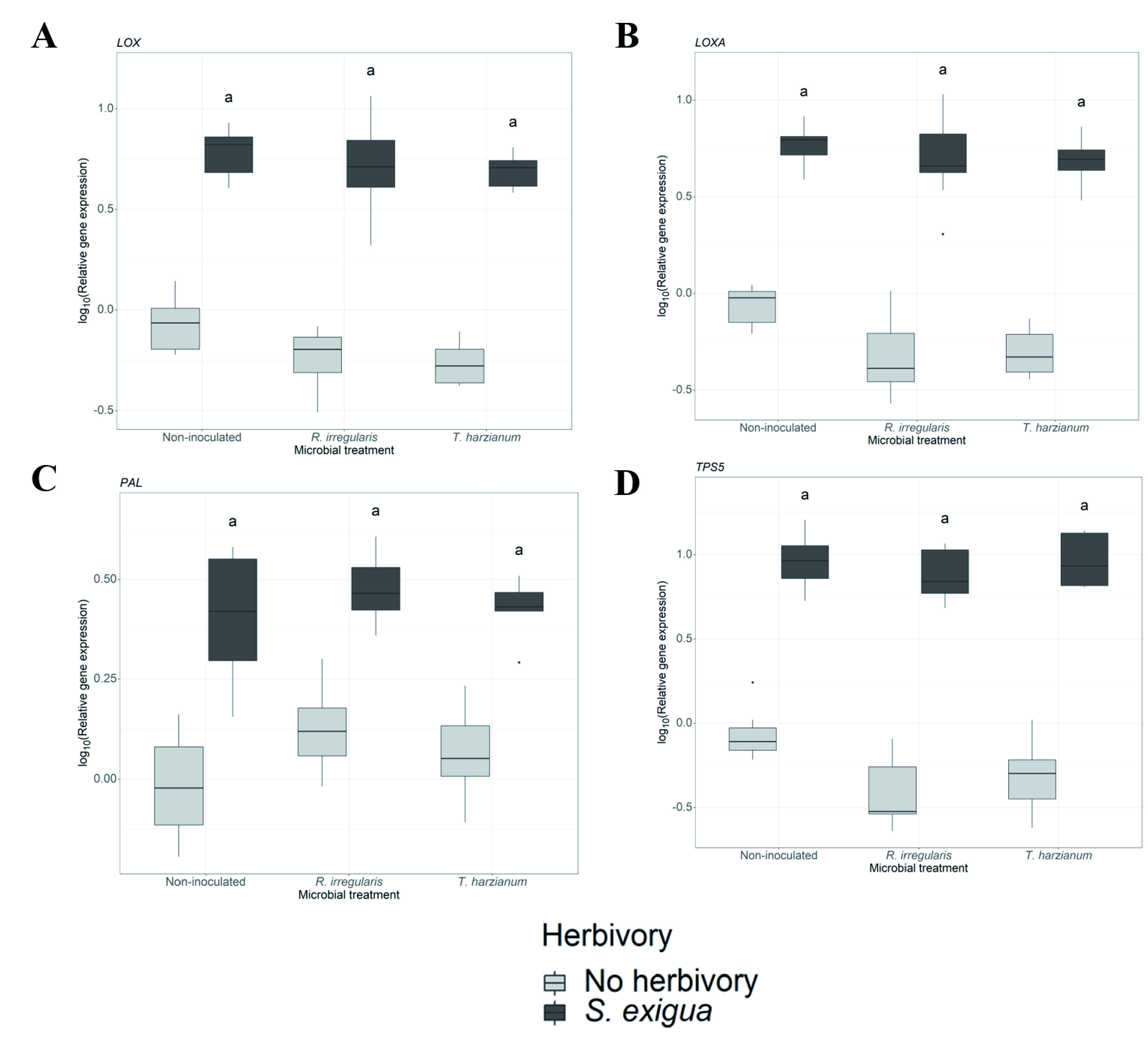

Supplement: Supplementary file 2 [file Image3.JPEG]

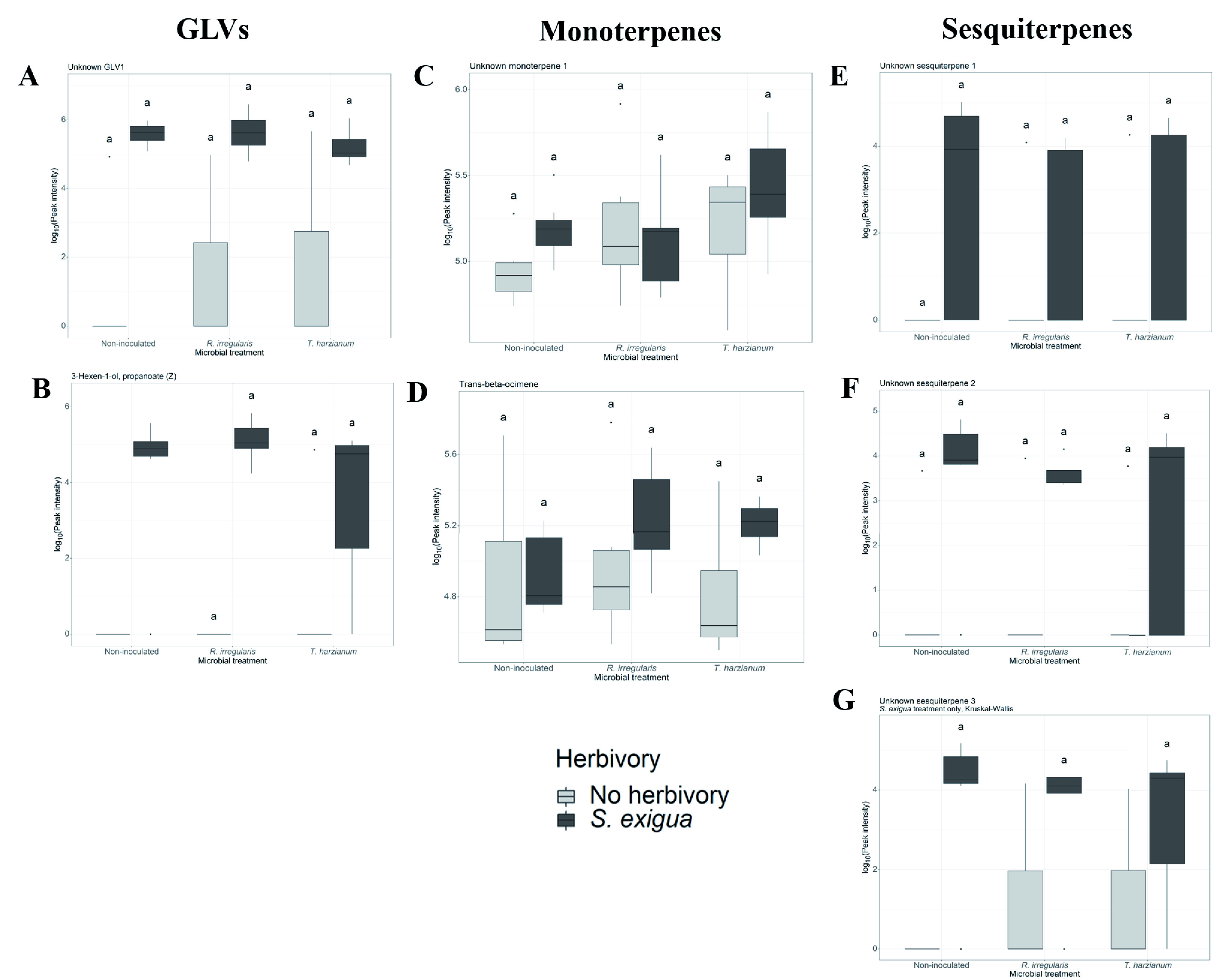

Supplement: Supplementary file 5 [file Image1.JPEG]

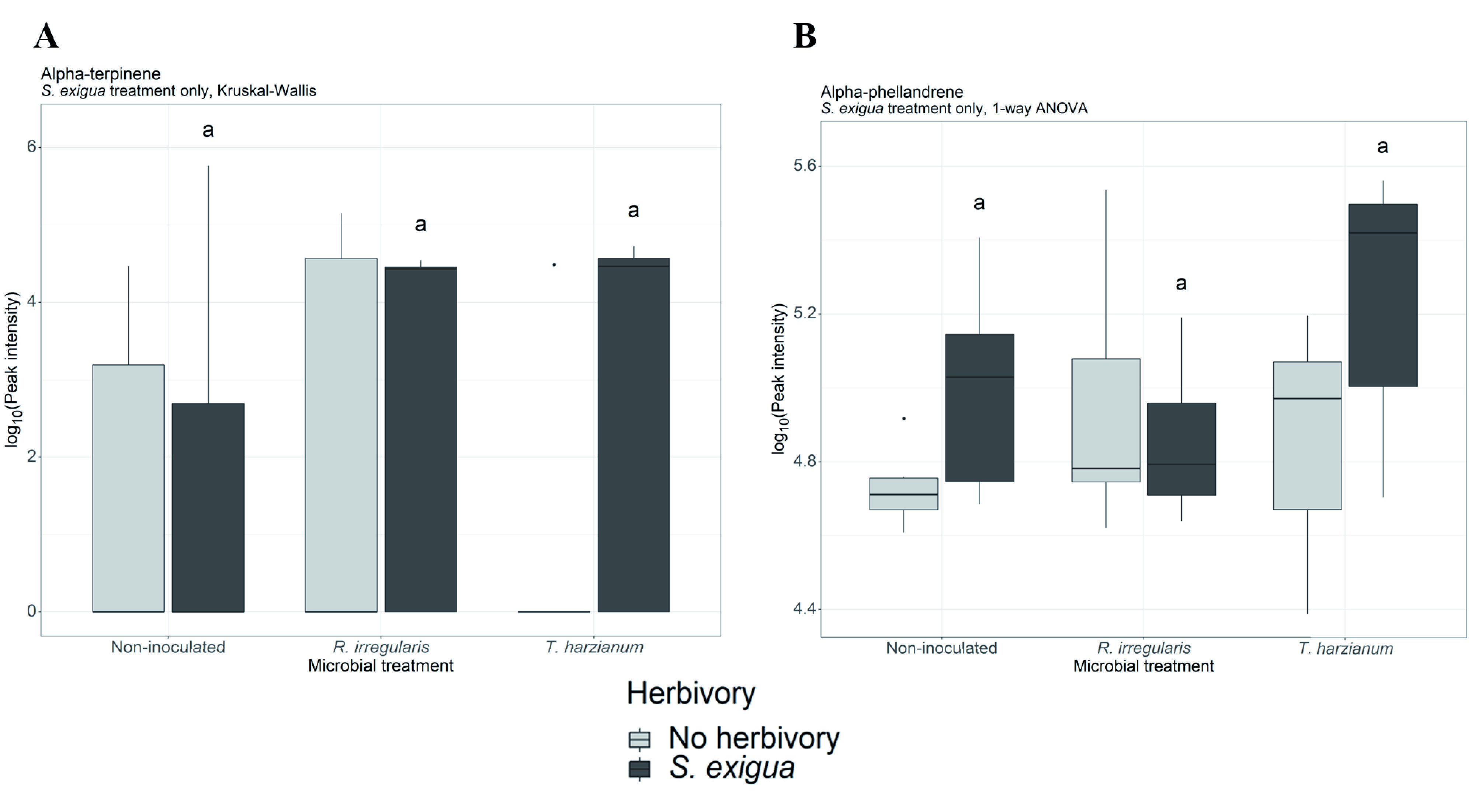

Supplement: Supplementary file 6 [file Image2.JPEG]
