# Supplementary material for: Root symbionts alter herbivore-induced indirect defenses of tomato plants by enhancing predator attraction
Source: Front Physiol. 2022 Oct 21;13:1003746. doi: 10.3389/fphys.2022.1003746 (PMC9634184; doi:10.3389/fphys.2022.1003746)
Supplement: Supplementary file 4 [file Table1.DOCX]

Supplementary Material

**Supplementary tables**

**Supplementary Table 1.** Statistical table for the volatiles analyzed with two-way analysis of variance (ANOVA).

| **Volatile compound** | **RT (min)** | **Two-way ANOVA** | | |
| --- | --- | --- | --- | --- |
|  |  | **Microbe (m)** | **Interaction (mxh)** | **Herbivore (h)** |
| *cis*-3-hexenylacetae | 3.770 | 0.101 | 0.484 | 0.002 (**) |
| unknown GLV1 | 4.080 | 0.631 | 0.105 | 0.271 |
| 3-hexen-1-ol, propanoate, (*Z*)- | 4.110 | 0.672 | NA | 0.99 |
| *cis*-3-hexenylbutyrate | 4.520 | 0.349 | 0.551 | 2.06e-05 (***) |
| *cis*-3-hexenyl isovalerate | 4.580 | 0.331 | 0.76 | 0.0001 (***) |
| methyl salicylate | 6.610 | 0.476 | 0.369 | 9.2e-10 (***) |
| unknown monoterpene 1 | 2.820 | 0.127 | 0.368 | 0.144 |
| limonene | 3.190 | 0.186 | 0.071 (.) | 0.023 (*) |
| *β*-phellandrene | 3.250 | 0.189 | 0.106 | 0.036 (*) |
| *trans*-*β*-ocimene | 3.430 | 0.574 | 0.349 | 0.025 (*) |
| unknown sesquiterpene 1 | 5.260 | 0.273 | 0.8 | 0.88 |
| caryophellene | 5.420 | 0.818 | 0.071 (.) | 4.72e-05 (***) |
| unknown sesquiterpene 2 | 5.640 | 0.086 (.) | 0.274 | 0.312 |
| *γ*-muurolene | 5.850 | 0.159 | 0.346 | 0.001 (**) |
| *α*-humulene | 5.880 | 0.983 | 0.2 | 0.0002 (***) |

**Supplementary Table 2.** Statistical table of the post hoc test for the volatiles analyzed with two-way analysis of variance (ANOVA).

| **Volatile** | **Treatment** | **Tukey post hoc** | | | | | |
| --- | --- | --- | --- | --- | --- | --- | --- |
|  |  | **Nm** | **Nm + h** | ***Rhi*** | ***Rhi* + h** | ***Th*** | ***Th* + h** |
| cis-3-hexenylacetate | Nm | - |  | NA | NA | NA | NA |
|  | Nm + h | NA | - | 0.14 | 0.99 | 0.48 | 0.85 |
|  | *Rhi* | NA |  | - | 0.999 | 0.99 | 0.476 |
|  | *Rhi* + h | NA |  | 0.99 | **-** | 0.03 | 0.66 |
|  | *Th* | NA |  |  |  | - |  |
|  | *Th* + h | NA |  | 0.47 | 0.66 | 0.24 | - |
| unknown GLV 1 | Nm | - |  | 0.99 |  | 0.98 |  |
|  | Nm + h | 0.04 | **-** | 0.04 | 0.99 | 0.59 | 0.99 |
|  | *Rhi* |  |  | **-** |  | 0.94 |  |
|  | *Rhi* + h | 0.03 |  | 0.003 | **-** | 0.47 | 0.97 |
|  | *Th* |  |  |  |  | **-** |  |
|  | *Th* + h | 0.07 |  | 0.006 |  | 0.68 | **-** |
| 3-hexen-1-ol, propanoate, (*Z*) | Nm | **-** |  | NA |  | NA |  |
|  | Nm + h | NA | **-** | NA | NA | NA | NA |
|  | *Rhi* |  |  | **-** |  | NA |  |
|  | *Rhi* + h | NA |  | NA | **-** | NA | NA |
|  | *Th* |  |  |  |  | **-** |  |
|  | *Th* + h | NA |  | NA |  | NA | **-** |
| cis-3-hexenylbutyrate | Nm | - |  | 0.999 |  | 0.984 |  |
|  | Nm + h | 0.048 | - | 0.04 | 0.996 | 0.598 | 0.999 |
|  | *Rhi* |  |  | - |  |  |  |
|  | *Rhi* + h | 0.032 |  | 0.003 | - | 0.471 | 0.976 |
|  | *Th* |  |  | 0.949 |  | - |  |
|  | *Th* + h | 0.07 |  | 0.006 |  | 0.682 | **-** |
| cis-3-hexenyl isovalerate | Nm | - |  | 0.99 |  | 0.99 |  |
|  | Nm + h | 0.54 | - | 0.06 | 0.99 | 0.12 | 0.9 |
|  | *Rhi* |  |  | - |  | 0.99 |  |
|  | *Rhi* + h | 0.53 |  | 0.07 | - | 0.12 | 0.93 |
|  | *Th* |  |  |  |  | - |  |
|  | *Th* + h | 0.25 |  | 0.01 |  | 0.02 | **-** |
| methyl salicylate | Nm | - |  | 0.99 |  | 0.99 |  |
|  | Nm + h | 0.0005 | - | 0.0009 | 0.99 | 0.0002 | 0.88 |
|  | *Rhi* |  |  | - |  | 0.99 |  |
|  | *Rhi* + h | 0.005 |  | 0.01 | - | 0.003 | 0.68 |
|  | *Th* |  |  |  |  | - |  |
|  | *Th* + h | 0.00002 |  | 0.00003 |  | 0.000009 | - |
| unknown monoterpene 1 | Nm | - |  | 0.6 |  | 0.64 |  |
|  | Nm + h | 0.69 | - | 0.99 | 0.99 | 0.99 | 0.65 |
|  | *Rhi* |  |  | - |  | 1 |  |
|  | *Rhi* + h | 0.9 |  | 0.99 | - | 0.99 | 0.54 |
|  | *Th* |  |  |  |  | - |  |
|  | *Th* + h | 0.06 |  | 0.7 |  | 0.7 | - |
| limonene | Nm | - |  | 0.5 |  | 0.83 |  |
|  | Nm + h | 0.28 | - | 0.99 | 0.89 | 0.91 | 0.65 |
|  | *Rhi* |  |  | - |  | 0.99 |  |
|  | *Rhi* + h | 0.92 |  | 0.98 | - | 0.99 | 0.17 |
|  | *Th* |  |  |  |  | - |  |
|  | *Th* + h | 0.01 |  | 0.4 |  | 0.15 | - |
| *β*-phellandrene | Nm | - |  | 0.61 |  | 0.82 |  |
|  | Nm + h | 0.34 | - | 0.99 | 0.89 | 0.94 | 0.8 |
|  | *Rhi* |  |  | - |  | 0.99 |  |
|  | *Rhi* + h | 0.95 |  | 0.98 | - | 0.99 | 0.22 |
|  | *Th* |  |  |  |  | - |  |
|  | *Th* + h | 0.02 |  | 0.44 |  | 0.24 | - |
| trans-*β*-ocimene | Nm | - |  | 0.99 |  | 0.99 |  |
|  | Nm + h | 0.99 | - | 0.99 | 0.63 | 0.97 | 0.6 |
|  | *Rhi* |  |  | - |  | 0.95 |  |
|  | *Rhi* + h | 0.62 |  | 0.67 | - | 0.25 | 0.99 |
|  | *Th* |  |  |  |  | - |  |
|  | *Th* + h | 0.59 |  | 0.63 |  | 0.21 | - |
| unknown sesquiterpene 1 | Nm | - |  | ΝΑ |  | ΝΑ |  |
|  | Nm + h | ΝΑ | - | 0.83 | 0.61 | 0.96 | 0.98 |
|  | *Rhi* |  |  | - |  | 0.99 |  |
|  | *Rhi* + h | ΝΑ |  | 0.99 | - | 0.99 | 0.9 |
|  | *Th* |  |  |  |  | - |  |
|  | *Th* + h | ΝΑ |  | 0.97 |  | 0.99 | - |
| caryophellene | Nm | - |  | 0.69 |  | 0.99 |  |
|  | Nm + h | 0.003 | - | 0.09 | 0.45 | 0.006 | 0.98 |
|  | *Rhi* |  |  | - |  | 0.88 |  |
|  | *Rhi* + h | 0.36 |  | 0.98 | - | 0.57 | 0.79 |
|  | *Th* |  |  |  |  | - |  |
|  | *Th* + h | 0.01 |  | 0.29 |  | 0.03 | - |
| unknown sesquiterpene 2 | Nm | - |  | 0.99 |  | 0.99 |  |
|  | Nm + h | 0.68 | - | 0.97 | 0.15 | 0.83 | 1 |
|  | *Rhi* |  |  | - |  | 0.99 | 0.99 |
|  | *Rhi* + h | 1 |  | 0.96 | - | 0.99 | 0.23 |
|  | *Th* |  |  |  |  | - |  |
|  | *Th* + h | 0.72 |  | 0.98 |  | 0.86 | - |
| *γ*-muurolene | Nm | - |  | 0.99 |  | 0.98 |  |
|  | Nm + h | 0.04 | - | 0.03 | 0.4 | 0.13 | 0.99 |
|  | *Rhi* |  |  | - |  | 0.99 |  |
|  | *Rhi* + h | 0.8 |  | 0.8 | - | 0.98 | 0.69 |
|  | *Th* |  |  |  |  | - |  |
|  | *Th* + h | 0.11 |  | 0.11 |  | 0.32 | - |
| *α*-humulene | Nm | - |  | 0.74 |  | 0.99 |  |
|  | Nm + h | 0.02 | - | 0.32 | 0.87 | 0.04 | 0.99 |
|  | *Rhi* |  |  | - |  | 0.92 |  |
|  | *Rhi* + h | 0.35 |  | 0.96 | - | 0.55 | 0.91 |
|  | *Th* |  |  |  |  | - |  |
|  | *Th* + h | 0.02 |  | 0.38 |  | 0.06 | - |

**Supplementary Table 3.** Statistical table for the volatiles analyzed with one-way analysis of variance (ANOVA).

| **Volatile compound** | **One-way ANOVA** | **Tukey HSD post hoc** | | |
| --- | --- | --- | --- | --- |
|  | **Microbe (m)** | ***Rhi* + h vs. Nm + h** | ***Th* + h vs. Nm + h** | ***Th* + h vs. *Rhi* + h** |
| *α*-phellandrene | 0.086 (.) | 0.727 | 0.248 | 0.086 (.) |

**Supplementary Table 4.** Statistical table for the volatiles analyzed with Kruskal-Wallis test.

| **Volatile compound** | **Kruskal-Wallis test** | **Dunn’s test** | | |
| --- | --- | --- | --- | --- |
|  | **Microbe (m)** | ***Rhi* + h vs. Nm + h** | ***Th* + h vs. Nm + h** | ***Th* + h vs. Rhi + h** |
| *α*-terpinene | 0.065 (.) | 0.058 (.) | 0.42 | 0.793 |
| unknown sesquiterpene 3 | 0.858 | 0.862 | 0.54 | 0.367 |

**Supplementary Table 5.** Statistical table for the expression analysis of the gene transcripts analyzed with two-way analysis of variance (ANOVA).

| **Gene** | **Two-way ANOVA** | | |
| --- | --- | --- | --- |
|  | **Microbe (m)** | **Interaction (m x h)** | **Herbivore (h)** |
| Allene oxide synthase 2 (*AOS2*) | 0.121 | 0.563 | 2.389e-10 (***) |
| Salicylic acid methyl transferase (*SAMT*) | 0.431 | 0.004 (**) | <2.2e-16 (***) |

**Supplementary Table 6.** Statistical table of the post hoc test for the expression analysis of the gene transcripts analyzed with two-way analysis of variance (ANOVA).

| **Gene** | **Treatment** | **Tukey post hoc** | | | | | |
| --- | --- | --- | --- | --- | --- | --- | --- |
|  |  | **Nm** | **Nm + h** | ***Rhi*** | ***Rhi* + h** | ***Th*** | ***Th* + h** |
| Allene oxide synthase 2 (*AOS2*) | Nm | - |  | 0.41 |  | 0.71 |  |
|  | Nm + h | 0.003 | - | 0.0000069 | 0.88 | 0.00002 | 1 |
|  | *Rhi* |  |  | - |  | 0.99 |  |
|  | *Rhi* + h | 0.06 |  | 0.0001 | - | 0.0006 | 0.88 |
|  | *Th* |  |  |  |  | - |  |
|  | *Th* + h | 0.004 |  | 0.00001 |  | 0.00003 | - |
| Salicylic acid methyl transferase (*SAMT*) | Nm | - |  | 0.12 |  | 0.07 |  |
|  | Nm + h | 0.0000036 | - | 0.0000001 | 0.99 | 0.0000001 | 0.29 |
|  | *Rhi* |  |  | - |  | 1 |  |
|  | *Rhi* + h | 0.00000006 |  | 0.00000001 | - | 0.00000001 | 0.49 |
|  | *Th* |  |  |  |  | - |  |
|  | *Th* + h | 0.0000001 |  | 0.00000001 |  | 0.0000001 | - |

**Supplementary Table 7.** Statistical table for the expression analysis of the gene transcripts analyzed with one-way analysis of variance (ANOVA).

| **Gene** | **One-way ANOVA** | **Tukey HSD post hoc** | | |
| --- | --- | --- | --- | --- |
|  | **Microbe (m)** | ***Rhi* + h vs. Nm + h** | ***Th* + h vs. Nm+ h** | ***Th* + h vs. *Rhi* + h** |
| Lipoxygenase 1 (*LOXA*) | 0.589 | 0.65 | 0.649 | 0.995 |
| Lipoxygenase (*LOX*) | 0.506 | 0.732 | 0.484 | 0.886 |
| Plenylalanine ammonia lyase (*PAL*) | 0.459 | 0.439 | 0.928 | 0.708 |

**Table 8.** Statistical table for the expression analysis of the gene transcripts analyzed with Kruskal-Wallis test.

| **Gene** | **Kruskal-Wallis test** | **Dunn’s test** | | |
| --- | --- | --- | --- | --- |
|  | **Microbe (m)** | **Nm + h vs. *Rhi* + h** | **Nm + h vs. *Th* + h** | ***Rhi* + h vs. *Th* + h** |
| Terpene synthase 5 (*TPS5*) | 0.2896 | 1 | 1 | 0.362 |

**Table 9.** Primer sequences used for the gene expression analysis of selected indirect defense-related genes.

| **Abbreviation** | **Target Gene** | **Sequence ID** | **Sequence (5’->3’)** |
| --- | --- | --- | --- |
| *SlEF* | Tomato elongation factor 1α | X14449.1 | Forward GATTGGTGGTATTGGAACTGTC  Reverse  AGCTTCGTGGTGCATCTC |
| *PAL* | *Solanum lycopersicum* phenylalanine ammonia-lyase 5 | NM_001320040.1 | Forward CGTTATGCTCTCCGAACATC  Reverse  GAAGTTGCCACCATGTAAGG |
| *SAMT* | *Solanum lycopersicum* S-adenosyl-L-methionine salicylic acid carboxyl methyltransferase | unknown | Forward  GGGTTGTTCTTCTGGAGCGA  Reverse  CGCGTTAAAATCATTTCCAGGGA |
| *LOX* | *Solanum lycopersicum* lipoxygenase (LOX1.1) | NM_001247927.2 | Forward  GGTTACCTCCCAAATCGTCC  Reverse  TGTTTGTAACTGCGCTGTG |
| *LOXA* | *Solanum lycopersicum* lipoxygenase (LOX1.1) | NM_001247927.2 | Forward  GGTTACCTCCCAAATCGTCC  Reverse  TGTTTGTAACTGCGCTGTG |
| *AOS2* | *Solanum lycopersicum* allene oxide synthase 2 (AOS2) | NM_001287778.2 | Forward  AGATTTTCTTCCCGAATATGCTGAA  Reverse  ATACTACTGATTTCATCAACGGCAT |
| *TPS5* | Monoterpene (Linalool) synthesis | AAX69063 | Forward  CTTCGGATGAACTGAAAAGAGG  Reverse  GTGGAGAATTTTTGCTTTGAGC |

**Table 10.** Relative gene expression levels ($2^{\text{–∆∆Ct}}$) of each treatment. The number of biological control used is shown as n under mean ± standard deviation.

| **Target gene** | **Non-inoculated** | ***R. irregularis*** | ***T. harzianum*** | **Non-inoculated+ *S. exigua*** | ***R. irregularis* + *S. exigua*** | ***T. harzianum* + *S. exigua*** |
| --- | --- | --- | --- | --- | --- | --- |
| *PAL* | 0.99 ± 0.11 (n=7) | 1.37 ± 0.15 (n=6) | 1.19 ± 0.11 (n=8) | 2.71 ± 0.3 (n=9) | 3.04 ± 0.19 (n=9) | 2.71 ± 0.14 (n=7) |
| *SAMT* | 1 ± 0.09 (n=7) | 0.64 ± 0.06 (n=6) | 0.64 ± 0.05 (n=8) | 2.81 ± 0.41 (n=8) | 2.85 ± 0.25 (n=9) | 3.75 ± 0.41 (n=7) |
| *LOX* | 0.88 ± 0.11 (n=7) | 0.6 ± 0.07 (n=6) | 0.55 ± 0.04 (n=8) | 6.28 ± 0.61 (n=8) | 5.86 ± 0.91 (n=9) | 4.95 ± 0.36 (n=7) |
| *LOXA* | 0.87 ± 0.07 (n=7) | 0.52 ± 0.1 (n=7) | 0.5 ± 0.04 (n=8) | 5.93 ± 0.49 (n=8) | 5.42 ± 0.83 (n=9) | 5.01 ± 0.58 (n=6) |
| *AOS2* | 1 ± 0.06 (n=7) | 0.8 ± 0.06 (n=7) | 0.87 ± 0.09 (n=8) | 1.56 ± 0.11 (n=9) | 1.4 ± 0.12 (n=9) | 1.54 ± 0.05 (n=8) |
| *TPS5* | 0.91 ± 0.14 (n=7) | 0.43 ± 0.08 (n=7) | 0.52 ± 0.09 (n=8) | 9.71 ± 1.29 (n=8) | 7.99 ± 0.97 (n=8) | 9.69 ± 1.2 (n=8) |

**Supplementary figures**

**Sup. Fig.1. Volatile compounds emitted by non-inoculated and root-inoculated tomato plants non-infested or infested by *Spodoptera exigua* larvae for twenty-four hours.** The compounds depicted belong to the class of green leaf volatiles (A, B), monoterpenes (C, D) and sesquiterpenes (E to G). Herbivory by *S. exigua* larvae had no significant effect on the emission of the compounds shown. Treatments are: control plants symbolized as Non-inoculated, *Rhizophagus irregularis*-root inoculated plants symbolized as *R. irregularis* and *Trichoderma harzianum* root-inoculated plants symbolized as *T. harzianum*. Light gray-colored boxplots represent non-herbivore-infested plants and dark gray-colored boxplots represent *S. exigua*-infested plants. Significant differences between treatments are indicated by different letters after Tukey’s *post hoc* tests after two-way ANOVA: *p* < 0.05. For the unknown sesquiterpene 3 (G), Kruskal-Wallis test was conducted only for the samples of *S. exigua*-infested plants.

**Sup. Fig. 2. Monoterpenes emitted by non-inoculated and root-inoculated tomato plants non-infested or infested by *Spodoptera exigua* larvae for twenty-four hours.** Root beneficial microbes marginally affected the emission levels of the compounds depicted. Treatments are: control plants symbolized as Non-inoculated, *Rhizophagus irregularis*-root inoculated plants symbolized as *R. irregularis* and *Trichoderma harzianum* root-inoculated plants symbolized as *T. harzianum*. Light gray-colored boxplots represent non-herbivore-infested plants and dark gray-colored boxplots represent *S. exigua*-infested plants. A Kruskal-Wallis test was conducted for alpha-terpinene and one-way ANOVA for alpha-phellandrene. Both Kruskal-Wallis test and one-way ANOVA were conducted only for the samples of *S. exigua*-infested plants.

**Sup. Fig. 3. Transcript levels of four defense-related genes in non-inoculated and root-inoculated tomato plants non-infested or infested by *Spodoptera exigua* larvae for twenty-four hours.** Herbivory by *S. exigua* larvae and root beneficial microbes did not affect the transcript levels of the genes shown. Values are the average of the three technical replicates for each of six-seven biological replicates per treatment. Expression levels for the depicted genes were normalized to the mean of tomato elongation factor 1a gene expression levels in each sample as reference. Light gray-colored boxplots represent non-herbivore-infested plants and dark gray-colored boxplots represent *S. exigua*-infested plants. One-way ANOVA was conducted for the genes *LOX*, *LOXA* and *PAL*. Kruskal-Wallis test was conducted for the gene *TPS5*. Both one-way ANOVA and Kruskal-Wallis test were conducted only for the samples of *S. exigua*-infested plants.
